# Identification and Characterization of a Novel Hepta-Segmented dsRNA Virus From the Phytopathogenic Fungus *Colletotrichum fructicola*

**DOI:** 10.3389/fmicb.2018.00754

**Published:** 2018-04-19

**Authors:** Lifeng Zhai, Meixin Zhang, Ni Hong, Feng Xiao, Min Fu, Jun Xiang, Guoping Wang

**Affiliations:** ^1^College of Life Science and Technology, Yangtze Normal University, Chongqing, China; ^2^National Key Laboratory of Agromicrobiology, Huazhong Agricultural University, Wuhan, China; ^3^College of Plant Science and Technology, Huazhong Agricultural University, Wuhan, China; ^4^Key Laboratory of Horticultural Plant Biology, Ministry of Education, Huazhong Agricultural University, Wuhan, China; ^5^Institute of Plant Quarantine, Chinese Academy of Inspection and Quarantine, Beijing, China

**Keywords:** Colletotrichum fructicola chrysovirus 1, tentative chrysovirus, hypovirulence, *Chrysoviridae*, genome, *Colletotrichum fructicola*, double-stranded RNA

## Abstract

A novel hepta-segmented double-stranded RNA (dsRNA) virus was isolated and characterized from the strain FJ-4 of the phytopathogenic fungus *Colletotrichum fructicola*, and was named Colletotrichum fructicola chrysovirus 1 (CfCV1). The full-length cDNAs of dsRNA1–7 were 3620, 2801, 2687, 2437, 1750, 1536, and 1211 bp, respectively. The 5′- and 3′-untranslated regions of the seven dsRNAs share highly similar internal sequence and contain conserved sequence stretches, indicating that they have a common virus origin. The 5′-and 3′-UTRs of the seven dsRNAs were predicted to fold into stable stem-loop structures. CfCV1 contains spherical virions that are 35 nm in diameter consisting of seven segments. The largest dsRNA of CfCV1 encodes an RNA-dependent RNA polymerase (RdRp), and the second dsRNA encodes a viral capsid protein (CP). The dsRNA5 encodes a C2H2-type zinc finger protein containing an R-rich region and a G-rich region. The smallest dsRNA is a satellite-like RNA. The functions of the other proteins encoded by dsRNA3, dsRNA4, dsRNA6 are unknown. Phylogenetic analysis, based on RdRp and CP, indicated that CfCV1 is phylogenetically related to Botryosphaeria dothidea chrysovirus 1 (BdCV1), and Penicillium janczewskii chrysovirus 2 (PjCV2), a cluster of an independent cluster II group in the family *Chrysoviridae*. Importantly, all the seven segments of CfCV1 were transmitted successfully to other virus-free strains with an all-or-none fashion. CfCV1 exerts minor influence on the growth of *C*. *fructicola* but can confer hypovirulence to the fungal host. To our knowledge, this is the first report of a hepta-segmented tentative chrysovirus in *C*. *fructicola*.

## Introduction

All major fungal groups, including yeast and filamentous fungi, are infected by mycoviruses (Pearson et al., 2009; Xie and Jiang, 2014; Ghabrial et al., 2015). The majority of mycoviruses possess either double-stranded (ds) or positive single-stranded (ss) RNA, but several have been found to possess DNA or negative-sense ssRNA as their genetic material (Yu et al., 2010; Liu et al., 2014; Ghabrial et al., 2015; Donaire et al., 2016; Marzano et al., 2016; Mu et al., 2018). The currently recognized dsRNA mycoviruses belong to six families, including *Totiviridae, Partitiviridae, Chrysoviridae, Megabirnaviridae, Quadriviridae*, and *Reoviridae* (Ghabrial and Suzuki, 2009; Xie and Jiang, 2014; Ghabrial et al., 2015). The taxonomy is regularly updated with the discovery of more novel mycoviruses (Xie and Jiang, 2014; Ghabrial et al., 2015).

Chrysoviruses possess four genomes that are separately encapsidated in virus particles and 2.4–3.6 kbp in length (Jiang and Ghabrial, 2004; Ghabrial and Suzuki, 2009). An RNA-dependent RNA polymerase (RdRp) is encoded by the largest dsRNA, while the other dsRNAs encodes a capsid protein (CP), a putative protease, and a protein with unknown function (Jiang and Ghabrial, 2004). Although the functions of proteins p3 and p4, respectively encode by dsRNA3 and dsRNA4, are unknown, the p3 sequence contains a “phytoreovirus S7 domain” with nucleic acid binding activities was found, and the p4 sequence contains motifs present in the conserved core of the ovarian tumor (OTU) gene-like superfamily (Ghabrial et al., 2015). The typical chrysoviruses were only found in filamentous fungi (Ghabrial et al., 2015). However, a group of viruses has been described as chrysovirus-related unclassified viruses which contain either three or five genome segments in the family *Chrysoviridae* (Darissa et al., 2011; Yu et al., 2011; Li et al., 2013; Urayama et al., 2014; Zhang et al., 2017). The chrysovirus-related unclassified viruses with three genomes have been isolated in plants and fungi (Li et al., 2013; Zhong et al., 2016; Zhang et al., 2017), while others with five genomes have been only isolated in fungi (Darissa et al., 2011; Yu et al., 2011; Urayama et al., 2014). Several of the fungi infected by chrysovirus-related unclassified viruses with five genomes exhibit abnormal symptoms, including mycelial growth delay and reduced fungal virulence (Urayama et al., 2010, 2014), whereas the reported typical chrysoviruses do not alter the phenotype of their hosts, with the exception of Botryosphaeria dothidea chrysovirus 1 (BdCV1), which was found in *Botryosphaeria dothidea* (Wang et al., 2014; Ghabrial et al., 2015; Ding et al., 2017).

Fungi of genus *Colletotrichum* are considered to be a highly economically significant pathogen and causes anthracnose disease in a variety of commercial crops (Cannon et al., 2012). *C. fructicola* causes leaf black spot and fruit rot disease in apple, pear, mango, citrus, grape (Liao et al., 2012; Vieira et al., 2014; Velho et al., 2015; Lima et al., 2015; Zhang et al., 2015). Mycoviruses infecting the members of *Colletotrichum* were isolated and characterized in recent years (de Figueirêdo et al., 2012; Zhong et al., 2014, 2016; Campo et al., 2016; Marzano et al., 2016; Rosseto et al., 2016; Jia et al., 2017). For example, an unidentified dsRNA virus with isometric viral-like particles was isolated from a *C. gloeosporioides* strain (de Figueirêdo et al., 2012). A partitivirus was previously characterized in the fungus *C*. *acutatum* (Zhong et al., 2014). Other mycoviruses in endophytic and phytopathogenic strains of *Colletotrichum* from different hosts have also been investigated (Campo et al., 2016; Marzano et al., 2016; Rosseto et al., 2016). Interestingly, a dsRNA virus with filamentous viral particles was isolated and characterized from *C*. *camelliae* (Jia et al., 2017). More recently, a tri-segmented chrysovirus-related unclassified virus was identified and characterized in a *C*. *gloeosprioides* strain (Zhong et al., 2016).

In this study, we report the isolation and characterization of a novel hepta-segmented dsRNA virus from a strain FJ-4 of the pear-infecting *C*. *fructicola* in China. Based on the viral genome organization, phylogeny and particle morphology, the novel virus was identified to belong to the chrysovirus-related unclassified viruses group in the family *Chrysoviridae*. Therefore, the virus was named Colletotrichum fructicola chrysovirus 1 (CfCV1). DsRNA1 and dsRNA 2 of CfCV1 encodes viral RdRp and CP, respectively. Interestingly, dsRNA5 of CfCV1 encodes a C2H2-type zinc finger protein. The smallest dsRNA is a satellite-like RNA. The functions of the proteins encoded by dsRNA3, dsRNA4, dsRNA6 are unknown. The CfCV1 exerts minor influence on the growth of *C*. *fructicola* but can confer hypovirulence to the fungal host. Taken together, our results demonstrate a novel hepta-segmented chrysovirus-related unclassified virus belong to chrysovirus cluster II in the *C*. *fructicola*.

## Materials and Methods

### Fungal Strains

Ten strains of *C*. *fructicola* were used in the present study (**Table 1**). Strain FJ-4 and virulent strain FJ-85 were isolated from diseased leaves of sandy pear (*Pyrus pyrifolia* Nakai cv. Cuiguan) collected in Fujian Province, China (Zhang et al., 2015). The strain FJ-4 was further used to extract dsRNA. The virus-free strain FJ-4-18, which is a single-conidium isolate of strain FJ-4, and the strain FJ-85^hyg^, which was labeled with a hygromycin resistance gene, were used for the horizontal transmission assay. Strains CDFJ418-1–3 from FJ-4-18 and CDFJ485-1–3 from FJ-85 were obtained from the horizontal transmission assay. Two out of 96 single-conidium sub-strains from the strain FJ-4, strain FJ-4-1 (virus-infected) and FJ-1-18 (virus-free), were using for biological tests as the control. All *C*. *fructicola* strains and their derivatives were stored at 4°C on potato dextrose agar (PDA) slants.

**Table 1:** Origin of strains of *Colletotrichum fructicola* used in this study.

Strain	Origin	Mycovirus	Source
FJ-4	*Pyrus pyrifolia*, Fujian, China	CfCV1-infected	This study
FJ-4-1	A single-conidium isolate of FJ-4	CfCV1-infected	This study
FJ-4-18	A single-conidium isolate of FJ-4	CfCV1-free	This study
FJ-85	*P. pyrifolia*, Fujian, China	CfCV1-free	Zhang et al., 2015
FJ-85^hyg^	FJ-85 labeled with a hygromycin resistance resistant gene	CfCV1-free	–^∗^
CDFJ418-1	FJ-4-18 in a pairing culture of FJ-4-18 and FJ-4	CfCV1-infected	This study
CDFJ418-2	FJ-4-18 in a pairing culture of FJ-4-18 and FJ-4	CfCV1-infected	This study
CDFJ418-3	FJ-4-18 in a pairing culture of FJ-4-18 and FJ-4	CfCV1-infected	This study
CDFJ485-1	FJ-85^hyg^ in a pairing culture of FJ-85^hyg^ and FJ-4	CfCV1-infected	This study
CDFJ485-2	FJ-85^hyg^ in a pairing culture of FJ-85^hyg^ and FJ-4	CfCV1-infected	This study
CDFJ485-3	FJ-85^hyg^ in a pairing culture of FJ-85^hyg^ and FJ-4	CfCV1-infected	This study

*^∗^The strain FJ-85^*hyg*^ was labeled by Zhang P. F. Biological features of strains FJ-85 and FJ-85^*hyg*^, including colony morphology, growth rate, and virulence, have not differentiated.*

### dsRNA Extraction and Purification

All *C*. *fructicola* strains evaluated in this study were cultured for 5 days on cellophane membranes placed on top of PDA plates. Upon harvesting, the mycelia were finely ground using liquid nitrogen, and a patented method was used to extract the dsRNA (Zhai et al., 2016). DNA contaminants and ssRNA were eliminated from the dsRNA preparation by digestion with DNase I and S1 nuclease (Takara, Dalian, China). The dsRNA in each extract was dissolved in diethypyrocarbonate (DEPC)-treated water and then visualized by electrophoresis on a 1.2% (w/v) agarose gel stained with 0.1 μg/ml ethidium bromide.

### Purification of Virus Particles From Mycelia

Differential centrifugation was used to isolate the virus-like particle (VLP) from the mycelia of strain FJ-4. The VLPs were then concentrated by sucrose density gradient centrifugation based on a modified protocol (Zhai et al., 2016), and then suspended in 0.05 M sodium phosphate buffer (pH = 7.4). Phosphotungstic acid (20 g/liter [wt/vol], pH = 7.4) staining was then used for observation of VLPs under a transmission electron microscope (TEM, H7650; Hitachi). Proteins and nucleic acids from the viral particles were analyzed as previously described (Zhai et al., 2016). The separated proteins in SDS-PAGE were used for polypeptide mass fingerprinting-mass spectrum (PMF-MS) analyses. After separation on a 1.2% (w/v) agarose gel, the viral dsRNAs were excised and purified using an AxyPrep^TM^ DNA Gel Extraction Kit (Axygen Scientific, Inc., Wujiang City, China). Following this they were dissolved in DEPC-treated water and used for cDNA cloning.

### CDNA Synthesis and Molecular Cloning

The method of CDNA libraries for the characterization of the dsRNA segments was constructed based on previous reports (Zhai et al., 2016). The obtained ds-cDNA insert segments (>0.5 kb) were used for nucleotide sequence analysis. Sequence gaps between clones were closed by reverse transcription (RT)-PCR using primers designed from the obtained cDNA sequences. The RLM-RACE procedure was conducted to obtain the terminal sequence of each of the dsRNAs as previously described (Liu et al., 2009). Briefly, Ligation of the 3′-terminus of each strand of dsRNA with the closed adaptor primer RACE-OLIGO [5′-(P)-GCATTGCATCATATCGATCGAATTCTTTAGTGAGGGTTAATTGCC-(NH2)-3′] was achieved using T4 RNA ligase (TaKaRa, Dalian, Ltd., China) at 8°C for 12 h. Reverse transcription of the oligonucleotide-ligated dsRNA was conducted using the M-MLV reverse transcriptase and oligo primer RACE-1st (5′-GGCAATTAACCCTCACTAAAG-3′). Amplification of the cDNA used another primer ORACE-2nd (5′-TCACTAAAGAATTCGATCGATC-3′) and the sequence-specific primers corresponding to the 5′- and 3′-terminal sequences of the dsRNAs, and LA Taq DNA polymerase. The resulting positive clones were sequenced at Genscript Biotechnology Co., Ltd., Nanjing, China, and the nucleotides were confirmed from no less than three independent, overlapping clones in both orientations.

### Sequence Analysis, Alignment, and Phylogenetic Analysis

The DNAMAN software package (DNAMAN version 6.0; Lynnon Biosoft, Montreal, QC, Canada) was used to detect potential open reading frame (ORF). Prediction of the stem-loop structures of the terminal sequences of the viral RNAs were conducted using the RNA folding program from the Mfold website, implementing the default parameters^1^ (Zuker, 2003). CD-search on the website of the National Center for Biotechnology Information (NCBI)^2^ and the motif scan website^3^ were used to identify the conserved domains of the full-length cDNA virus sequences. MAFFT software (Katoh and Standley, 2013) was used for multiple nucleotide and amino acid sequence alignments, and the results were visualized using BoxShade^4^. Phylogenetic trees were constructed using the maximum likelihood method in Molecular Evolutionary Genetics Analysis (MEGA) software 7 (Kumar et al., 2016). Reference sequences of the viruses used for comparative analyses were obtained from the NCBI database^5^ (Supplementary Table S1).

### Vertical and Horizontal Transmission Assays

Single-conidium sub-strains from the strain FJ-4 were used for evaluating the vertical transmission of mycovirus. Conidia from strain FJ-4 were induced on PDA plate as a previously described (Zhang et al., 2015). The individual sub-strains were obtained as described previously (Zhai et al., 2016), and 96 individual single-conidium sub-strains were assessed for the presence of dsRNA segments in the mycelia using previously described methods (Zhai et al., 2016). Horizontal transmission of dsRNA segments via hyphal anastomosis was executed according to the previously described method (Zhang and Nuss, 2008). The strain FJ-4 served as the donor, and strains FJ-85^hyg^ and FJ-4-18, which lacked detectable dsRNAs (**Table 1**), served as the recipients. Each mycovirus-free strain and the strain FJ-4 were co-cultured on the same PDA plates in three replicates. After 5 days of co-incubation at 25°C, mycelial agar disks were obtained from the edge of the recipient strain that was most distant from the contact point between the two colonies. To exclude for the co-presence of the parental FJ-4 in the sub-strains, the obtained strains were then cultured on PDA containing 60 μg/mL hygromycin following 5 days. The newly acquired strains were cultured onto a fresh PDA without hygromycin. Furthermore, three derivatives from strain FJ-85^hyg^ (CDFJ-485-1, CDFJ-485-2, and CDFJ-485-3) were individually purified by single hyphal tip culturing and used to assess the mycovirus content and biological properties. Another assay was implemented to explore the length of time when the virus transmitted from strain FJ-4 to FJ-85^hyg^. Strains were co-cultured on PDA plates as described above with four replicates, and mycelial agar disks were cut out at 24, 48, and 72 h. Seven specific primers were used for detections of dsRNA1–7 (Supplementary Table S2).

### Biological Tests

Using previously described procedures (Zhai et al., 2016), the mycelial growth rate, colony morphology, and virulence of virus-infected and -free strains of *C*. *fructicola* were assessed (**Table 1**). The strain FJ-4-1 but not all single-conidium sub-strains harbored dsRNAs was used as a control in this experiment. Briefly, mycelial growth rates of those strains were measured on fresh PDA plates at 25°C. Each of the strains has three replicates. To assay the virulence of those strains, actively growing mycelial plugs from each strain were inoculated onto four detached fruit of pear (*P*. *bretschneideri* cv. Huangguan). Inoculated fruits were maintained in a 25°C incubator for 6 days and then average lesion size was measured for analysis. The biological properties were investigated by one-way analysis of variance using the SAS 9.0 program and *P*-values < 0.05 were considered statistically significant.

## Results

### A Complex dsRNA Pattern in Strain FJ-4

**FIGURE 1:**
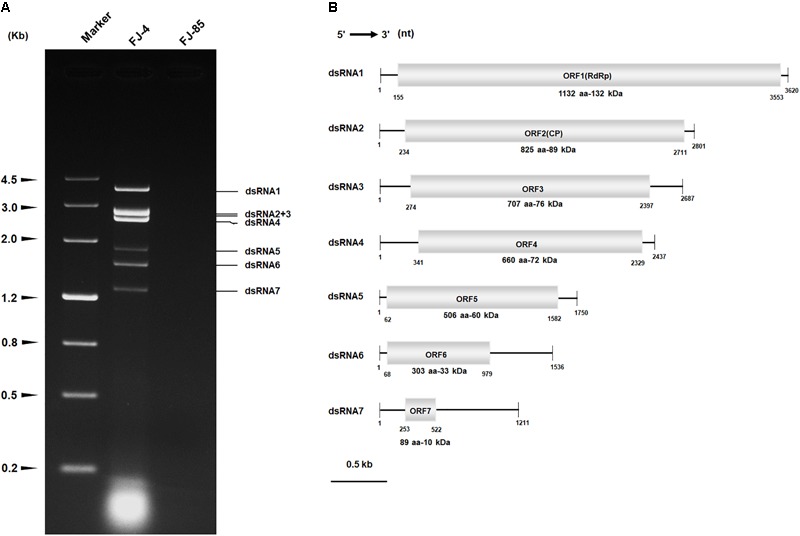
Double-stranded RNA (dsRNAs) extracted from strain FJ-4 and the genomic organization of Colletotrichum fructicola dsRNA virus 1 (CfCV1). **(A)** 1.2% agarose gel electrophoretic profiles of dsRNA preparations extracted from strains FJ-4 and FJ-85 after digestion with DNase I and S1 nuclease. **(B)** Genomic organization of dsRNA1–7 of CfCV1.

The dsRNAs were extracted from the mycelia of strain FJ-4 and analyzed by agarose gel electrophoresis. Following digestion of the strain preparations with DNase I and S1 nuclease, a complex pattern with six bands from 1.3 to 4.0 kb was observed (**Figure 1A**). In subsequent experiments, the results of ds-cDNA library clones revealed that the second band comprised two different dsRNAs. Therefore, the seven dsRNAs were termed dsRNA1–7. No dsRNA was detected in the preparations from strain FJ-85 under the same treatment conditions (**Figure 1A**).

### Nucleotide Sequencing Analysis of Seven dsRNA

Each of the seven dsRNA complete nucleotide sequences was acquired from the ds-cDNA library clones, gap-filling RT-PCR clones, and RACE clones. The full-length cDNAs of dsRNA1–7 were 3620, 2801, 2687, 2437, 1750, 1536, and 1211 bp, respectively (**Figure 1B**). The corresponding sequences were deposited in GenBank under accession numbers MG425969–MG425975. BLASTn searches revealed that the dsRNA1 sequence shared low similarity (*E*-value 9e-49 to 1e-20; 10–37% coverage; 65–70% identity) with the dsRNA1 of the four chrysoviruses including BdCV1, Penicillium janczewskii chrysovirus 1 (PjCV1), PjCV2, and Aspergillus mycovirus 1816 (AsV1816). Furthermore, the dsRNA 2–7 sequences were highly dissimilar to the other viral RNA sequences.

Sequence analysis of the full-length dsRNA1 cDNA indicated a size of 3620 bp with a GC content of 54.5%. A single large ORF from positions 155 nt to 3553 nt was discovered and this ORF encodes a putative protein (P1) of 1132 amino acid residues with a mass of approximate 132 kDa (**Figure 1B**). Based on the scanning motifs, P1 was found to be most similar to the viral RdRp family (Pfam02123, RdRp_4), and BLASTp analysis indicated that P1 was closely related to the putative RdRp of chrysoviruses/“Chryso-like” viruses with 23 to 49% identity (Supplementary Table S3). In addition, two bipartite nuclear localization signal profiles from amino acid 988 to 1005 (RRVRVIGKMRVPKVKAKL, *E* value = 2.1e-04) and from 1053 to 1067 (RRLLARRICOKRGKV, *E* value = 2.1e-04) were also found in the C-terminal region of the protein P1.

The full-length cDNA of dsRNA 2 was 2801 bp in length with a GC content of 57.8%, and also harbored a single ORF on the genomic plus strand RNA. The protein (P2) encoded by dsRNA2 ORF was determined to have a molecular mass of 89 kDa (**Figure 1B**). A BLASTp search of P2 showed significant similarities to putative of some chrysoviruses/“Chryso-like” viruses with 27–36% identities, including PjCV2-P2, BdCV1-P2, PjCV1-P2, Fusarium oxysporum f. sp. dianthi mycovirus 1-P3 (FodCV1-P3), and Fusarium graminearum mycovirus-China 9-P3 (FgV-ch9-P3), Magnaporthe oryzae chrysovirus 1-A (MoCV1-A-P4), and MoCV1-B-P4 (Supplementary Table S4). Interestingly, a conserved domain that has a sequence similarity with staphylocoagulase repeat domain (position 323–333; *E*-value = 0.097) was detected in the CfCV1-P2 protein.

An assessment of the full-length cDNA of dsRNA 3 indicated that it was 2687 bp in length with a GC content of 56.1%. The protein (P3) encoded by dsRNA3 ORF was determined to have a molecular mass of 76 kDa (**Figure 1B**). A BLASTp search of P3 showed significant similarity to the proteins BdCV1-P3, PjCV1-P3, PjCV2-P3, MoCV1-A-P2, and MoCV1-B-P2 with 26–31% identities (Supplementary Table S4).

Based on analysis of the full-length cDNA, dsRNA 4 was 2437 bp in length with a GC content of 56.7%. The protein (P4) encoded by dsRNA4 ORF had a molecular mass of 72 kDa (**Figure 1B**). A BLASTp search of P4 indicated significant similarity to the proteins BdCV1-P4, PjCV1-P4, PjCV2-P4, MoCV1-A-P3, MoCV1-B-P3, FodCV1-P2, Fusarium graminearum dsRNA mycovirus-2-P2 (FgV2-P2), and FgV-ch9-P2 with 30–40% identities (Supplementary Table S4). The motifs (I–IV) of the ovarian tumour (OUT) domain peptidase, which are yet to be determined of the other typical chrysoviruses with four segments (Covelli et al., 2004; Jamal et al., 2010), were not found in P4 of CfCV1.

**FIGURE 2:**
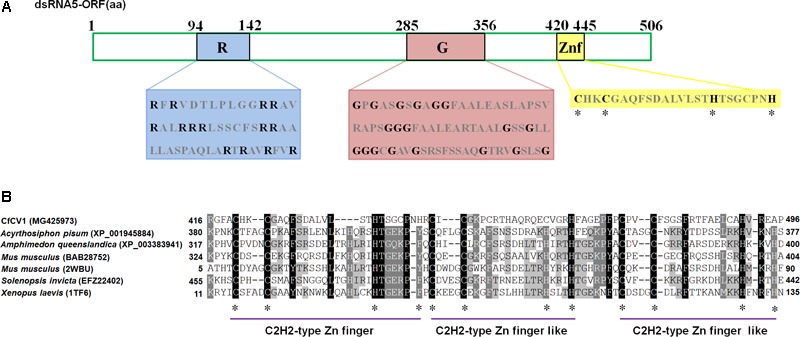
The diagrammatic C2H2-type zinc finger protein. **(A)** The zinc finger protein with an arginine-rich region (94–142) and a glycine-rich region (285–256). The arginine (R) and glycine (G) residues were indicated by black letters, and other amino acid residues were shown by gray letters. **(B)** Multiple sequence alignment of zinc finger protein in CfCV1 and some animals. The alignments were produced in MAFFT and visualized using the BoxShade program. “^∗^” indicates identical cysteine and histidine residues.

The full-length cDNA of dsRNA5 was 1750 bp in length and had a GC content of 60.5%. Furthermore, a single ORF was found in one of the strands of this dsRNA that encodes a 60 kDa protein (P5) (**Figure 1B**). BLASTp searches of the deduced amino acid sequences of P5 revealed the highest sequence similarity (identity 34%, *E*-value 0.027) with a C2H2-type zinc finger protein of *Oncorhynchus mykiss*. This zinc finger domain of P5 possessed a consensus sequence of CX_2_CX_12_HX_6_H at amino acids 420–445 (**Figures 2A,B**). There was an arginine-rich (R-rich) region (position 94–142) in the protein, accounting for 28.0% of the entire coverage of the R-rich region (**Figure 2A**). A glycine-rich (G-rich) region (position 285–356) was also found in P5, accounting for 26.0% of the entire coverage of the G-rich region (**Figure 2A**). However, other C2H2-type zinc finger -like regions (CXCX_12_CX_3_H and CX_2_CX_13_HX_3_C) were also found in the C-terminal regions (**Figure 2B**). The C2H2 zinc finger domains were also found at the C-terminus of the protein P5 encoded by dsRNA5 of FgV-ch9 while CfCV1-P5 and FgV-ch9-P5 had not similarity (Darissa et al., 2011; Yu et al., 2011).

dsRNA6 was revealed to be 1536 bp in length with a GC content of 57.1%. It contained a single ORF encoding a 33 kDa protein (P6) (**Figure 1B**). However, sequence analysis showed no known protein with a significant similarity to dsRNA6 ORFs-encoded protein. Sequence analysis revealed that dsRNA7 was 1211 bp in length and had a GC content of 54.7%. Examination for the presence of ORFs in the dsRNA7 only revealed in the strands a short ORF potentially encoding polypeptides of a size 10.0 kDa (**Figure 1B**). Furthermore, the potential sequence relationship between dsRNA7 and others of CfCV1 was not observed. Therefore, the dsRNA7 is not indispensable for virus CfCV1, and it might be a satellite-like RNA.

### 5′- and 3′-Untranslated Regions (UTRs)

**FIGURE 3:**
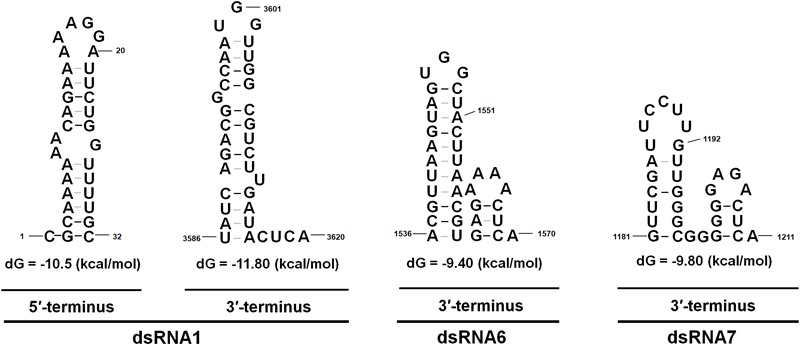
The proposed secondary structures with the lowest energy of CfCV1. The 5′ terminus of the coding strands of the seven dsRNAs were predicted to fold into semblable stem-loop structures, as depicted for dsRNA1. Whereas, the stem-loop structures of the 3′ terminus of dsRNA6 and dsRNA7 differed from that of dsRNA 1–5.

The 5′- UTR of the coding strands of dsRNA1–7 were 154, 233, 273, 340, 61, 67, and 252 nt in length, respectively (**Figure 1B**). High sequence similarity was detected based on a direct comparison of the nucleotide sequences of the 5′-UTR of the seven dsRNA segments (Supplementary Figures S1A–C). In addition to the completely conserved 5′ termini (positions 1–18, AGCAAAAAACAGAAAAAAG), a UUUU region with a high sequence identity occurred within the 5′-UTR of all the seven dsRNAs (Supplementary Figure S1A). Differently, 24 nt of the dsRNA1–4, and 16 nt of the dsRNA5–7 were strictly conserved (Supplementary Figures S1B,C). The (CAA)n repeats discovered in the 5′-UTRs of all the dsRNA segments in typical chrysoviruses were detected in dsRNA1–4 of CfCV1 (Supplementary Figure S1A). The 3′-UTR of the coding strands of dsRNAs 1–7 were 67, 90, 290, 109, 168, 557 and 689 nt in length, respectively (**Figure 1B**). Conversely, at the 3′ end, 5 nt (ACUCA) of the dsRNA1–7 were conserved. Within the 3′-UTR, 6 nt (UAUCAG) and 11 nt (CUUGAUACUCA) of the dsRNA1–4, and 16 nt (AGGUUCGAUUCCU) and 5 nt (ACUCA) of the dsRNA5–7 were strictly conserved (Supplementary Figures S1A–C). The 5′-UTRs of the seven dsRNAs were predicted to fold into stable stem-loop structures, as depicted for dsRNA1 (**Figure 3**). However, the 3′-UTRs of five dsRNAs (dsRNA1–5) were predicted to fold into similar stem-loop structures, as illustrated in dsRNA1 (**Figure 3**). Whereas, the stem-loop structures of the 3′-UTRs of dsRNA6 and dsRNA7 were different (**Figure 3**).

### Phylogenetic Analysis of CfCV1

**FIGURE 4:**
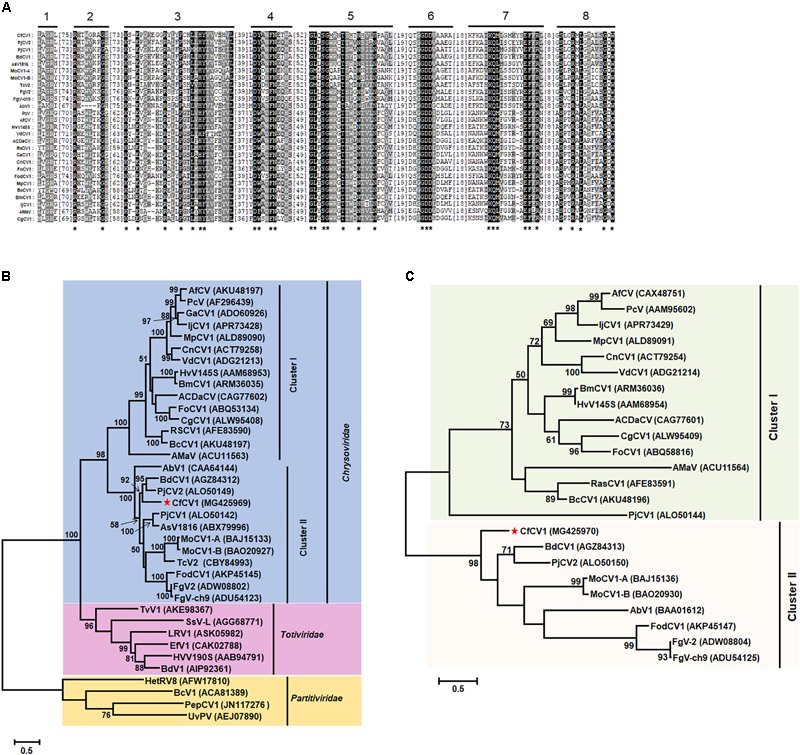
Phylogenetic analysis of CfCV1. **(A)** Multiple sequence alignments of RdRp in CfCV1 and other members of the family *Chrysoviridae*. The alignments were produced in MAFFT and visualized using the BoxShade program. “^∗^” indicates identical amino acid residues. **(B)** Maximum likelihood phylogenetic tree constructed using the complete amino acid sequences of the RdRp of CfCV1 and other selected *Chrysoviridae, Partitiviridae*, and *Totiviridae* members. **(C)** Maximum likelihood phylogenetic tree constructed using the complete amino acid sequences of the CP of CfCV1 and other selected members of the family Chrysoviridae. The red star indicates the position of CfCV1. The trees were computed based on the best-fit model of protein evolution (LG + G + I + F). The Gamas values were 3 (RdRp) and 2 (CP). Bootstrap values (relative) generated using 1000 replicates are shown on the branches. Only ≥50% bootstrap values are shown, and branch lengths are proportional to the genetic distances. Virus name abbreviations and GenBank accession numbers are provided in Supplementary Table S1.

Multiple amino acid alignments of the predicted RdRp indicated the existence of the motifs I-VIII in CfCV1 and other members of the family *Chrysoviridae* (**Figure 4A**). A maximum-likelihood phylogenetic tree was constructed based on the amino acid sequences of RdRp encoded by some dsRNA viruses. The phylogenetic tree indicated that the members of the family *Chrysoviridae* clustered two distinct clusters (**Figure 4B**; Ghabrial et al., 2018). Cluster I contained the members of the genus *Chrysovirus* and 3-segmented chrysovirus-related unclassified viruses (**Figure 4B**). CfCV1 was clustered together with PjCV2 and BdCV1 to form a separated evolutionary clade in the cluster II which contained chrysovirus-related, unclassified viruses with four or five genomic segments (**Figure 4B**; Ghabrial et al., 2018). In the case of the RdRp-based phylogenetic tree, the amino acid sequence of RdRps from selected members of the families *Totiviridae* and *Partitiviridae* were also included (**Figure 4B**). Phylogenetic analysis, based on the complete amino acid sequence of the CP of selected members of the family *Chrysoviridae*, was performed (**Figure 4C**). The results indicated that CfCV1 was also clustered together with PjCV2 and BdCV1. The CP-based phylogenetic tree was generated and showed similar to that constructed from the RdRp alignment (**Figure 4C**). Therefore, CfCV1 may be a new member of cluster II virus in the family *Chrysoviridae*.

### Virus Particle

**FIGURE 5:**
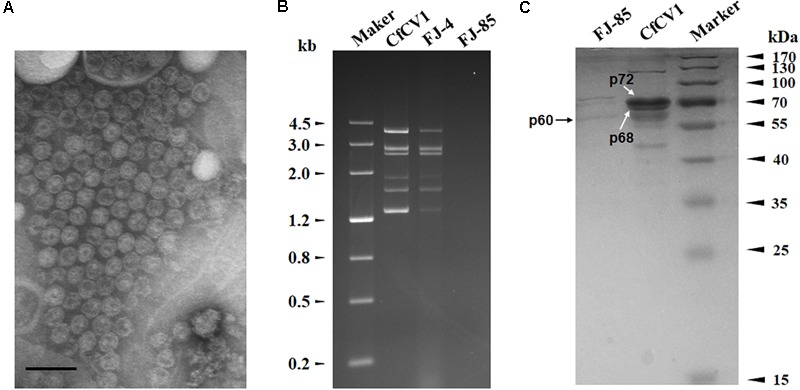
VLP composition of CfCV1. **(A)** TEM observation of the VLPs. **(B)** 1.2% agarose gel electrophoresis in TAE buffer for 16 h at 50 V of the dsRNA profiles from mycelia and VLPs. **(C)** 10% SDS-PAGE gel-electrophoresis analysis of the protein components of the purified VPs from CfCV1-infected strain (lane CfCV1). The arrows showed the 60, 68, and 72 kDa proteins. The VLPs purified from the strain FJ-85 were analyzed using the same method (lane FJ-85). The protein markers were used to estimate the molecular weight of the protein bands. The scale bar represents 50 nm.

Virus particles were purified from the mycelia of strain FJ-4 using sucrose gradient centrifugation. Agarose gel electrophoresis of the nucleic acids extracted from the 10–50% sucrose gradient fractions (at 10% sucrose increments) showed that the dsRNA segments of CfCV1 were mostly recovered from the 30% fraction (Supplementary Figure S2). The result suggested that VLP fractions containing dsRNA were detected in agarose gels and dsRNA1–7 were detected in the same fraction. To examine the morphology of VLPs of CfCV1, The fraction containing viral dsRNAs were centrifuged and re-suspended. The VLPs purified from strain FJ-4 of *C*. *fructicola* were isometric and approximately 35 nm in diameter as observed under TEM (**Figure 5A**). Similar migration rates of dsRNAs from the purified VLPs were found to those directly extracted from the mycelia of strain FJ-4 (**Figure 5B**), and two major protein bands were detected with approximate sizes of 72 kDa (p72) and 68 kDa (p68) (**Figure 5C**, lane CfCV1) in the suspension containing VLPs from the virus-infected strain FJ-4. The preparations from the virus-free strain FJ-85, based on an identical method to that used for the virus-infected strain, did not appear to contain these proteins (**Figure 5C**, lane FJ-85). A 60 kDa (p60) protein was identified in both the virus-free and virus-infected preparations, and was likely to be a host protein that was co-fractionated with the virus particles (**Figure 5C**).

### Analysis of Virus Structural Proteins

The CfCV1 virus particle preparations revealed two specific protein bands (**Figure 5C**). These were individually assessed and their corresponding genes determined using PMF-MS. A total of 43 and 34 peptide fragments were discovered in p72 and p68, respectively (Supplementary Tables S5, S6). The 72 kDa protein matched the partial sequence at amino acid 1–766 of P2 encoded by dsRNA2, accounting for 88.7% of the entire coverage (852 amino acids). The 34 peptides from p68 corresponded to an ORF4-encoded protein at the amino acid position of 2–560, accounting for 83.3% of the complete coverage (660 amino acids). Based on the PMF-MS results, p72 and p68 were confirmed to correspond to the deduced 89- and 72-kDa proteins encoded by the ORFs of dsRNA2 and dsRNA4, respectively (**Figure 5C**, lane CfCV1).

### Vertical and Horizontal Transmission of the dsRNA Virus

Totally, ninety-six single-conidium strains were derived from strain FJ-4. Detection of dsRNAs revealed that a strain FJ-4-18 was not infected by CfCV1 and the others harbored seven dsRNA segments. This result revealed that CfCV1 in strain FJ-4 could be vertically transmitted to the single-conidium sub-strains. The dsRNA-free strains FJ-58^hyg^ and FJ-4-18 were used as the recipients in the horizontal transmission assay. For each strain, three derivative sub-strains were obtained in this assay (**Table 1**). The seven dsRNAs were recovered in derivatives from the recipient strains. The presence of the viral dsRNAs in these derivatives was confirmed by RT-PCR using the primers specific for the CfCV1 dsRNA1 and sequencing of the amplified products (data not show). CfCV1 from strain FJ-4 was therefore horizontally transmitted to the other strains via hyphal contact. Interestingly, it only took 48 h for CfCV1 to infect the strain FJ-85^hyg^ from the strain FJ-4 via hyphal contact (Supplementary Figure S3). The result revealed that dsRNA1–7 of CfCV1were co-transit at the same time (Supplementary Figure S3). In the co-cultivation, strains FJ-4 and FJ-85 exhibited an obvious boundary compared to strain FJ-4 and FJ-4-18 (Supplementary Figure S4A). However, in the boundary part, the induced apoptosis was not being observed at the hyphal tips of strains FI-4 and FJ-85 (Supplementary Figure S3B). Strain FJ-4 showed sparser hypha than CfCV1-free strains FJ-4-18 and FJ-85 (Supplementary Figure S4B).

### Influence of CfCV1 on Biological Properties of *C*. *fructicola*

**FIGURE 6:**
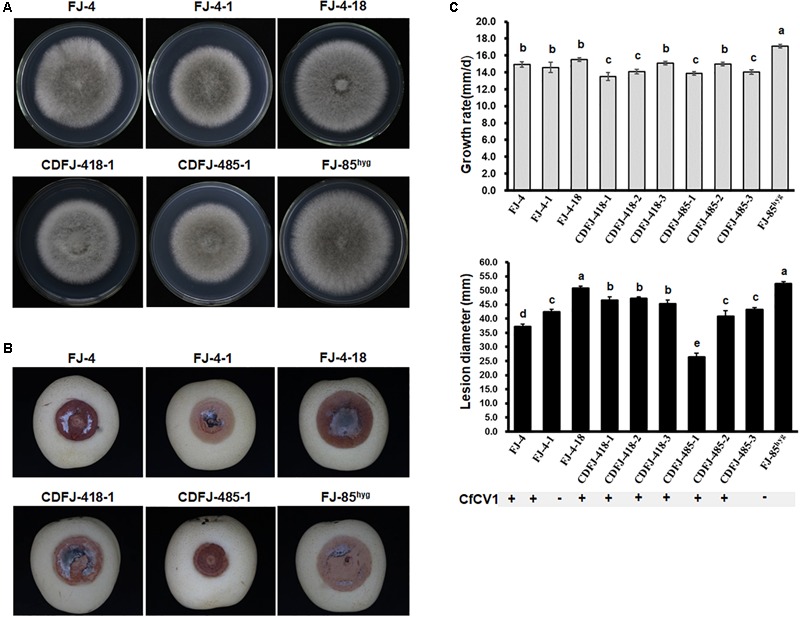
Colony morphology and virulence of strain FJ-4, strain FJ-85 and derived sub-strains on pear fruit. **(A)** Colony morphology in PDA medium (25°C, 4 days). **(B)** Pear fruits wound-inoculated with colonized plugs of tested strains, and photographed at 6 days post inoculation. **(C)** Statistical analysis of the growth grate and the lesion size. The error bars indicate the SD from three sample means. The letter indicates a significant difference at the *P* < 0.05 level of confidence according to multiple range test.

Two CfCV1-free and eight CfCV1-infected strains were subjected to biological assessment, including colony morphology, growth rate, and virulence. On PDA plates, the colonies of all tested strains possessed radially growing and thick hyphae (**Figure 6A**). The average of growth rates of each tested strains were shown to vary between 13.5 and 17.0 mm/d. The averages of growth rates of CfCV1-infected strains FJ-4 (14.9 mm/day), FJ-4-1 (14.6 mm/day) and CDFJ-418-3 (15.1 mm/day) were similar to that of CfCV1-free strain FJ-4-18 (15.5 mm/day), which was an isogenic fungal strain from FJ-4 (**Figure 6C**). Whereas, the growth rates of CDFJ-418-1 (13.5 mm/day) and CDFJ-418-2 (14.1 mm/day) were significantly lower than that of strain FJ-4-18 (**Figure 6C**). Compared to the growth rate of the strain FJ-85^hyg^ (17.1 mm/day), derivatives CDFJ-485-1(13.9 mm/day), CDFJ-485-2 (15.0 mm/day), and CDFJ-485-3(14.0 mm/day) infected by CfCV1 showed slower growth (**Figure 6C**). These results revealed that the CfCV1 exerted minor influence on the growth rate of *C*. *fructicola*. A pathogenicity test on pear fruit for 6 days revealed that all the tested strains caused rot lesions on the pear fruit. The average diameter of lesions induced by FJ-4-18 (50.9 mm) was significantly larger than those induced by the isogenic fungal strains FJ-4 (37.2 mm), FJ-4-1 (42.4 mm), CDFJ-418-1 (46.6 mm/d), CDFJ-418-2 (47.2 mm) and CDFJ-418-3 (45.2 mm) (**Figure 6B**). Consistently, the smallest lesion was observed in CDFJ485-1 (25.0 mm) and the diameters of lesions lesion induced by CDFJ485-1, CDFJ485-2, and CDFJ485-3 were significantly smaller than those caused by their mother strain FJ-85^hyg^ (**Figures 6B,C**). Therefore, CfCV1 can confer hypovirulence to the fungal host *C*. *fructicola*.

## Discussion

The molecular and biological characteristics of the hepta-segmented dsRNA virus CfCV1 during infection of the plant fungal pathogen *C*. *fructicola* were investigated in this study. Phylogenetic analysis indicated that CfCV1 was more closely related to members of cluster II in the family *Chrysoviridae*, and had high amino acid sequence similarity to PjCV2. However, there are some unique properties that are distinct from members of family *Chrysoviridae*.

### Genome Organization and Characteristics

In this study, a cluster II chrysovirus (CfCV1) with seven segments was described. Members of the family *Chrysoviridae* possess different genome segment numbers ranging from three to five (Darissa et al., 2011; Yu et al., 2011; Li et al., 2013; Urayama et al., 2014). Typical members of the genus *Chrysovirus* contain four dsRNA elements (Jiang and Ghabrial, 2004; Ghabrial et al., 2015). Furthermore, an increasing number of studies have shown that some dsRNA viruses possessing three or five genomic dsRNA components display evolutionary features similar to those of chrysoviruses (Urayama et al., 2010; Yu et al., 2011; Darissa et al., 2011; Li et al., 2013; Urayama et al., 2014; Zhong et al., 2016). They were named chrysovirus-related unclassified viruses (Ghabrial et al., 2018). Cluster I contained the members of the genus *Chrysovirus* and 3-segmented chrysovirus-related unclassified viruses, and cluster II contained chrysovirus-related, unclassified viruses with four or five genomic segments (Ghabrial et al., 2018).

CfCV1 contained seven dsRNA elements, despite the fact that dsRNA5–7 contain ORFs with no detectable identity with those of other viruses based on the available sequences. Sequences at the 5′- and 3′-UTRs are highly conserved among the dsRNA segments of members of the family *Chrysoviridae* (Darissa et al., 2011; Yu et al., 2011; Li et al., 2013; Urayama et al., 2014; Wang et al., 2014; Ghabrial et al., 2015). The dsRNA1–7 of CfCV1 share a similar conserved 5′- and 3′-terminal sequence, 18 nt (AGCAAAAAACAGAAAAAAG) at 5′ end and 5 nt (ACUCA) at 3′ end (see Supplementary Figure S1). The conserved terminal sequences of viral genomic RNA are generally involved in the packaging of viruses (Anzola et al., 1987; Wei et al., 2003). There were (CAA)n repeats at the 5′-UTRs of the genomic dsRNA1–4, which is characteristic of typical members of this genus *Chrysovirus* (Jiang and Ghabrial, 2004; Ghabrial et al., 2015). However, it was absent in the genomic RNA5–7. The (CAA)n repeats were also found at the 5′-UTRs of FgV2 which was belong to chrysovirus cluster II group (Darissa et al., 2011).

In the phylogenetic trees, based on RdRps and CPs amino acid sequences, CfCV1 was clustered together with BdCV1 and PjCV2, which contain four dsRNAs elements (Wang et al., 2014; Nerva et al., 2016; Ding et al., 2017), and other chrysovirus cluster II viruses, including MoCV1-A, MoCV1-B, FgV2, and FgV-ch9, which contain five dsRNAs elements. Therefore, CfCV1 is a new tentative member of the family *Chrysoviridae*. We also observed that members of the family *Chrysoviridae* were clustered into two main groups (chrysovirus cluster I and chrysovirus cluster II groups) in the phylogenetic tree based on RdRp and CP sequences (see **Figure 4B**; Ghabrial et al., 2018). The chrysovirus cluster I group maintained classified members of the genus *Chrysovirus* combined with the tri-segmented chrysovirus-related viruses, including Anthurium mosaic-associated virus (AmAV), Brassica campestris chrysovirus 1 (BcCV1), Raphanus sativus chrysovirus 1 (RsCV1), and Colletotrichum gloeosporioides chrysovirus 1 (CgCV1). However, some chrysovirus-related viruses with four dsRNA elements were found in the chrysovirus cluster II group. It was revealed that these viruses in possession of different numbers of segments might constitute the evolutionary ancestors (Darissa et al., 2011; Yu et al., 2011; Li et al., 2013; Urayama et al., 2014). Mycoviruses containing a variable number of genomic segments were also described in polymycoviruses having at least four and up to eight genomic segments (Kanhayuwa et al., 2015; Zhai et al., 2016; Kotta-Loizou and Coutts, 2017; Jia et al., 2017). Therefore, chrysovirus cluster II group viruses in the family *Chrysoviridae* might have a variable number of genomic segments.

Based on the results of proteins from the viral particles using PMF-MS and SDS-PAGE, p72 and p68 were confirmed to correspond to the proteins encoded by the ORFs of dsRNA2 and dsRNA4, respectively. It has been suggested that the capsids of the chrysovirus cluster II viruses appear to contain more than one structural protein, as described in BdCV1 and MoCV1-A (Urayama et al., 2012; Wang et al., 2014). The conserved core of the OTU gene-like superfamily of predicted proteases were found in many typical chrysoviruses (Jamal et al., 2010; Cao et al., 2011; Ghabrial et al., 2015), but multiple alignments showed that motifs of those were absent in the ORFs of CfCV1 (data not shown). Even though a protein of five segmented chrysovirus-related virus (FgV2-P3) contained motifs III and IV in the OTU proteins was described (Yu et al., 2011), the motif PGDGXCXXHX, along with motifs II (with a conserved K), III and IV (with a conserved H), form the conserved core of the OTU gene-like superfamily of predicted proteases (Makarova et al., 2000; Covelli et al., 2004; Jamal et al., 2010).

### The Possible Biological Function of dsRNA5–7

CfCV1 contained seven dsRNA elements, relating to the typical chrysoviruses, additional dsRNA5–7 contain ORFs with no detectable identity with those of other viruses. The conserved 5′- and 3′-terminal sequence detected between 5′- and 3′-terminal regions of the dsRNA1–7 of CfCV1 suggested that they had a common ancestor (Covelli et al., 2008). Our results showed that P5 encoded by dsRNA5 of CfCV1was a zinc finger protein. Interestingly, the N-terminal regions of CfCV1-P5 contained an R-rich region and a G-rich region. Furthermore, the C2H2 zinc finger domain also presented at the C-terminus of the protein encoded by dsRNA5 of FgV-ch9 and FgV2 (Darissa et al., 2011; Yu et al., 2011). The physiological functions of zinc finger protein in the biological processes of CfCV1 and FgV-ch9 remain largely unknown (Darissa et al., 2011; Yu et al., 2011). Future studies on detailed biological functions of CfCV1-P5 should be provided. The function of CfCV1-P6 and CfCV1-P7 remained largely unknown. DsRNA7 of CfCV1 might be a satellite-like RNA. Some small dsRNAs (570–610 bp in length) were also found in ACDaCV (Covelli et al., 2008). DsRNA7 of CfCV1 was 1211 bp in length, and it might longer than that of other satellite RNAs. However, no major ORFs were found in it and a lack of significant sequence similarity in databases. A large satellite-like RNA (1647 bp in length) also was found in Sclerotinia sclerotiorum botybirnavirus 1 (SsBRV1), and it might play some roles in modulating the virulence of the virus (Liu et al., 2015). It is noteworthy that the satellite-like RNA (dsRNA 3) of SsBRV1 can be readily removed via sub-culturing (Liu et al., 2015). However, the dsRNA1–7 of CfCV1 co-infected the fungus *C*. *fructicola* in vertical and horizontal transmission assays. Particularly, a single-spore strain without this dsRNA7 segment was not found among the 96 single-conidium strains detected. All the seven segments of CfCV1 were horizontally or vertically transmitted in an all-or-none fashion. These results implied that the dsRNA5–7 may play important roles in biological functions used by CfCV1. Further studies are required to determine whether a novel genome packaging mechanism or a protein subunit arrangement is involved. On the other hand, like other chrysovirus transfection (Wang et al., 2014), in the transfection assays with purified VLPs, CfCV1 particles were unsuccessfully transmitted to strain FJ-85 (data not show). Nevertheless, it will be interesting to investigate how the dsRNA5–7 of CfCV1interacts with its associated host.

### Relationship With Other Chrysoviruses

In the phylogenetic tree, CfCV1 is clustered together with BdCV1 and PjCV2, which contain four dsRNAs elements and belong in chrysovirus cluster II group (Wang et al., 2014; Nerva et al., 2016; Ghabrial et al., 2018). BLASTp analysis indicated that the P2–P4 proteins was closely related to the corresponding proteins of BdCV1, PjCV1, PjCV2, MoCV1-A, MoCV1-B, FgV2, and FgV-ch9 (see Supplementary Table S4). In addition, the P5 protein of CfCV1 was not similarity with any proteins of MoCV1-A, MoCV1-B, FgV2, and FgV-ch9 which contains five dsRNAs elements (Urayama et al., 2010, 2014; Darissa et al., 2011; Yu et al., 2011). CfCV1 might be closer tetra-segment than quin-segment members in the evolution in chrysovirus cluster II virus group. However, the 5′-termini of all dsRNAs of members of family *Chrysoviridae* contain a conserved sequence (Ghabrial et al., 2015). Particularly, 24 nt (AGCAAAAAACAGAAAAAGGAUUCU) of CfCV1 has a high sequence similarity to 24 nt (GCAAAAAAGAGAAUAAAGCUUUCU) of MoCV1-B (Urayama et al., 2014). On the other hand, ORF4 of typical chrysoviruses encode an OTU protein (Jiang and Ghabrial, 2004; Ghabrial et al., 2015), but it was not found in P4 of CfCV1. The OTU protein was rarely described in chrysovirus cluster II viruses (Urayama et al., 2010, 2014; Darissa et al., 2011; Yu et al., 2011; Wang et al., 2014). The result of multiple alignments showed that the motifs of OTU protein were not found in the ORFs of CfCV1 and other chrysovirus cluster II viruses (data not show). The (CAA)n repeats at the 5′-UTRs of the genomic dsRNAs were characteristic of typical members of this genus *Chrysovirus* (Jiang and Ghabrial, 2004). The repeats were also noticed in the 5′-UTRs of dsRNA1–4 of CfCV1 (see ??) and dsRNA1–5 of FgV-ch9 (Darissa et al., 2011). However, it was absent in the genomic dsRNAs of other members of chrysovirus cluster II group. Although the RdRps encoded by typical chrysoviruses have the P7/P-loop NTPase domain at their N-termini (Jiang and Ghabrial, 2004; Jamal et al., 2010; Ghabrial et al., 2015), dsRNAs of CfCV1 encoded proteins lack the motifs. Therefore, the CfCV1 detected from *C. fructicola* strain FJ-4 is probably a new member in the chrysovirus cluster II virus based on its genome sequence and structures.

### Biological Effect of CfCV1

In the horizontal transmission experiments with contact culture, CfCV1 from strain FJ-4 could infect FJ-85^hyg^ of *C. fructicola*. This positive result revealed that the virus could overcome the vegetative incompatibility between the different strains. CfCV1 was successfully transmitted from strain FJ-4 into FJ-4-18, an isogenic fungus with strain FJ-4. In many studies, mycoviruses were successfully transmitted into isogenic fungus, but not into strains with different origins, as proposed before for BdCV1 (Wang et al., 2014). Many chrysoviruses have been identified to infect phytopathogenic fungi, but few of them have been involved in the hypovirulence of their host fungi (Xie and Jiang, 2014; Ghabrial et al., 2015). However, BdCV1, a chrysovirus cluster II virus, appeared to alter colony morphology and reduce the virulence of their host fungi (Wang et al., 2014; Ding et al., 2017). CfCV1 and BdCV1 share similarly taxonomical relationship. There is a significant difference in the biological effects on the fungal hosts between CfCV1 and other chrysoviruses. BdCV1 leads to seriously debilitating symptoms in its fungal host *B*. *dothidea* (Wang et al., 2014; Ding et al., 2017). CfCV1 has no obvious effects on colony morphology (see **Figure 6A**) but has more hypovirulence than CfCV1-free strains on the detached fruit (see **Figures 6B,C**). Infection by CfCV1 induced no obvious changes in growth of *C. fructicola* strains, but it resulted in significant weakling of virulence. In addition, the rot lesions induced by sub-strain CDFJ-485-1 were half of those induced by his parental strain FJ-85. Moreover, MoCV1-A and MoCV1-B did not show a very severe symptom to the host fungus (Urayama et al., 2010, 2014). It was possible that dsRNAs 5–7 suppress the negative effects on hosts of dsRNAs 1–4 as a possibility. The results further supported the suggestion that CfCV1 is closely associated with the hypovirulence of the phytopathogenic fungus. The CfCV1-infected fungal strains maintained active growth. The feature is similar to those of the chrysoviruses, which do not alter the phenotype of their hosts. Although CfCV1 was not a good candidate for the biological control of anthracnose disease, the CfCV1-infected strains can be used for exploring the pathogenic mechanism of *C. fructicola*.

## Conclusion

We characterized the biological and molecular features of a hepta-segmented dsRNA virus that infects *C. fructicola*. The CfCV1 belongs to a chrysovirus cluster II taxonomic group in the family *Chrysoviridae*. CfCV1 has spherical virions that are 35 nm in diameter. The largest dsRNA of CfCV1 encodes an RNA-dependent RNA polymerase (RdRp), and the second dsRNA encodes a viral capsid protein (CP). The dsRNA5 encodes a C2H2-type zinc finger protein containing an R-rich region and a G-rich region. The smallest dsRNA is a satellite-like RNA. The functions of the proteins encoded by dsRNA3, dsRNA4, and dsRNA6 are unknown. CfCV1 can be transmitted vertically via asexual spores, or horizontally to other strains of *C. fructicola* via hyphal contact. Importantly, all the seven segments of CfCV1 were horizontally or vertically transmitted in an all-or-none fashion. Sub-strains infected by CfCV1 exhibited weak growth, virulence, and a sectoring phenotype. To the best of our knowledge, this is the first report of a hepta-segmented chrysovirus cluster II virus infected fungus of *C*. *fructicola*.

## Author Contributions

LZ, NH, and GW: conceived and designed the experiments. LZ, MZ, and JX: performed the experiments and analyzed the data. LZ and MZ: wrote the paper. LZ, MZ, FX, and MF: prepared the tables and figures. LZ, NH, and GW: revised and approved the final version of the paper. This is the first submission of this manuscript, and it is not being considered for publication elsewhere in part or in whole. All authors approved the submission of this manuscript.

## Conflict of Interest Statement

The authors declare that the research was conducted in the absence of any commercial or financial relationships that could be construed as a potential conflict of interest.

## Footnotes

1 http://mfold.rna.albany.edu/?q=mfold/RNA-Folding-Form2.3

2 http://www.ncbi.nlm.nih.gov/

3 http://www.genome.jp/tools/motif/

4 http://www.ch.embnet.org/software/BOX_form.html

5 http://www.ncbi.nlm.nih.gov/genomes
