# Supplementary material for: Identification and Characterization of a Novel Hepta-Segmented dsRNA Virus From the Phytopathogenic Fungus Colletotrichum fructicola
Source: Front Microbiol. 2018 Apr 19;9:754. doi: 10.3389/fmicb.2018.00754 (PMC5917037; doi:10.3389/fmicb.2018.00754)
Supplement: Supplementary file 1 [file Table_1.DOCX]

**Supplementary**

**Table S1.** Viral isolates used for sequence alignment and phylogenetic analysis of their RdRps.

| Viruses | Abbreviation | Host | Genus | Family | GenBank accession no. | | |
| --- | --- | --- | --- | --- | --- | --- | --- |
|  |  |  |  |  | RdRp |  | CP |
| Colletotrichum fructicola chrysovirus 1 | CfCV1 | Fungus | “Chryso-like” | *Chrysoviridae* | MG425969 | MG425969 | |
| Tolypocladium cylindrosporum virus 2 | TcV2 | Fungus | “Chryso-like” | *Chrysoviridae* | CBY84993 | - | |
| Magnaporthe oryzae chrysovirus 1-A | MoCV1-A | Fungus | “Chryso-like” | *Chrysoviridae* | BAJ15133 | BAJ15136 | |
| Magnaporthe oryzae chrysovirus 1-B | MoCV1-B | Fungus | “Chryso-like”*s* | *Chrysoviridae* | BAO20927 | BAO20930 | |
| Fusarium graminearum dsRNA mycovirus-2 | FgV2 | Fungus | “Chryso-like” | *Chrysoviridae* | ADW08802 | ADW08804 | |
| Fusarium graminearum mycovirus-China 9 | FgV-ch9 | Fungus | “Chryso-like” | *Chrysoviridae* | ADU54123 | ADU54125 | |
| Fusarium oxysporum f. sp. dianthi mycovirus 1 | FodCV1 | Fungus | “Chryso-like” | *Chrysoviridae* | AKP45145 | AKP45147 | |
| Agaricus bisporus virus 1 | AbV1 | Fungus | “Chryso-like” | *Chrysoviridae* | CAA64144 | BAA01612 | |
| Raphanus sativas chrysovirus 1 | RSCV1 | Plant | “Chryso-like” | *Chrysoviridae* | AFE83590 | AFE83591 | |
| Brassica campestris chrysovirus 1 | BcCV1 | Plant | “Chryso-like” | *Chrysoviridae* | AKU48197 | AKU48196 | |
| Colletotrichum gloeosporioides chrysovirus 1 | CgCV1 | Fungus | “Chryso-like” | *Chrysoviridae* | ALW95408 | ALW95409 | |
| Botryosphaeria dothidea chrysovirus 1 | BdCV1 | Fungus | *Chrysovirus* | *Chrysoviridae* | AGZ84312 | AGZ84313 | |
| Penicillium janczewskii chrysovirus 1 | PjCV1 | Fungus | *Chrysovirus* | *Chrysoviridae* | ALO50142 | ALO50144 | |
| Penicillium janczewskii chrysovirus 2 | PjCV2 | Fungus | *Chrysovirus* | *Chrysoviridae* | ALO50149 | ALO50150 | |
| Aspergillus mycovirus 1816 | AmV1816 | Fungus | *Chrysovirus* | *Chrysoviridae* | ABX79996 | - | |
| Verticillium dahliae chrysovirus 1 | VdCV1 | Fungus | *Chrysovirus* | *Chrysoviridae* | ADG21213 | ADG21214 | |
| Cryphonectria nitschkei chrysovirus 1 | CnCV1 | Fungus | *Chrysovirus* | *Chrysoviridae* | ACT79258 | ACT79254 | |
| Helminthosporium victoriae 145S virus | HvV145S | Fungus | *Chrysovirus* | *Chrysoviridae* | AAM68953 | AAM68954 | |
| Amasya cherry disease associated chrysovirus | ACDaCV | Plant | *Chrysovirus* | *Chrysoviridae* | CAG77602 | CAG77601 | |
| Bipolaris maydis chrysovirus 1 | BmCV1 | Fungus | *Chrysovirus* | *Chrysoviridae* | ARM36035 | ARM36036 | |
| Aspergillus fumigatus chrysovirus | AfCV1 | Fungus | *Chrysovirus* | *Chrysoviridae* | CAX48749 | CAX48751 | |
| Penicillium chrysogenum virus | PcV | Fungus | *Chrysovirus* | *Chrysoviridae* | AF296439 | AAM95602 | |
| Grapevine associated chrysovirus-1 | GaCV1 | Fungus | *Chrysovirus* | *Chrysoviridae* | ADO60926 | - | |
| Anthurium mosaic-associated virus | AMaV | Plant | *Chrysovirus* | *Chrysoviridae* | ACU11563 | ACU11564 | |
| Fusarium oxysporum chrysovirus 1 | FoCV1 | Fungus | *Chrysovirus* | *Chrysoviridae* | ABQ53134 | ABQ58816 | |
| Macrophomina phaseolina chrysovirus 1 | MpCV1 | Fungus | *Chrysovirus* | *Chrysoviridae* | ALD89090 | ALD89091 | |
| Isaria javanica chrysovirus 1 | IjCV1 | Fungus | *Chrysovirus* | *Chrysoviridae* | APR73428 | APR73429 | |
| Beet cryptic virus 1 | BcV1 | Plant | *Alphapartitivirus* | *Partitiviridae* | ACA81389 | - | |
| Heterobasidion partitivirus 8 | HetRV8 | Fungus | *Betapartitivirus* | *Partitiviridae* | AFW17810 | - | |
| Ustilaginoidea virens partitivirus 1 | UvPV1 | Fungus | *Gammapartitivirus* | *Partitiviridae* | AGO04402 | - | |
| Pepper cryptic virus 1 | PepCV1 | Plant | *Deltapartitivirus* | *Partitiviridae* | AEJ07890 | - | |
| Scheffersomyces segobiensis virus L | SsV-L | Fungus | *Totivirus* | *Totiviridae* | AGG68771 | - | |
| Helminthosporium victoriae virus 190S | HvV190S | Fungus | *Victorivirus* | *Totiviridae* | AAB94791 | - | |
| Botryosphaeria dothidea victorivirus 1 | BdV1 | Fungus | *Victorivirus* | *Totiviridae* | AIP92361 | - | |
| Epichloe festucae virus 1 | EfV1 | Plant | *Victorivirus* | *Totiviridae* | CAK02788 | - | |
| Trichomonas vaginalis virus 1 | TvV1 | Animal | *Trichomonasvirus* | *Totiviridae* | AKE98367 | - | |
| Leishmania RNA virus 1 | LRV1 | Animal | *Leishmaniavirus* | *Totiviridae* | ASK05982 | - | |
