# Supplementary material for: Identification and Characterization of a Novel Hepta-Segmented dsRNA Virus From the Phytopathogenic Fungus Colletotrichum fructicola
Source: Front Microbiol. 2018 Apr 19;9:754. doi: 10.3389/fmicb.2018.00754 (PMC5917037; doi:10.3389/fmicb.2018.00754)
Supplement: Supplementary file 2 [file Table_2.DOCX]

**Supplementary**

**Table S2.** Oligonucleotide primers used for dsRNA1–7 sequences amplification in this study.

| Name | Sequence (5′→ 3′) | Position | Size of the product |
| --- | --- | --- | --- |
| dsRNA1F | CGTCTACATCCACACGGTCGAG | dsRNA-1: 508–529 | 1035 bp |
| dsRNA1R | GGCGTTATCCTGTCCTGCATTTCG | dsRNA-1: 1542–1519 |  |
| dsRNA2F | CTGGACAACGTCGCAAAAGC | dsRNA-2: 885–904 | 899 bp |
| dsRNA2R | CCAGCGAAGTGCCTAATGTG | dsRNA-2: 1783–1764 |  |
| dsRNA3F | GGTGAGGAGCTGGTCGTAGAG | dsRNA-3: 523–543 | 580 bp |
| dsRNA3R | ACCCTTCCAAAACGTTGAGACC | dsRNA-3:1081–1102 |  |
| dsRNA4F | TACCGTTCCCTCCTGACG | dsRNA-4: 1001–1018 | 414 bp |
| dsRNA4R | GTAGGGTAGTATCGCTTCTGATG | dsRNA-4: 1414–1391 |  |
| dsRNA5F | CCGCTTTGCTCGCTTCTCC | dsRNA-5: 429–447 | 421 bp |
| dsRNA5R | TCGGTACGCAACACAGGAG | dsRNA-5:849–831 |  |
| dsRNA6F | CGCCAAAAGTACTGCAACCTC | dsRNA-6: 453–473 | 433 bp |
| dsRNA6R | GCCTCAATAACACGGACGTACC | dsRNA-6: 885–864 |  |
| dsRNA7F | CGGCTGTTAGTGTTTCACCAC | dsRNA-7: 174–194 | 808 bp |
| dsRNA7R | CCAGCAGGTATCATCGTCG | dsRNA-7: 981–963 |  |
