# Supplementary material for: Identification and Characterization of a Novel Hepta-Segmented dsRNA Virus From the Phytopathogenic Fungus Colletotrichum fructicola
Source: Front Microbiol. 2018 Apr 19;9:754. doi: 10.3389/fmicb.2018.00754 (PMC5917037; doi:10.3389/fmicb.2018.00754)
Supplement: Supplementary file 3 [file Table_3.DOCX]

**Supplementary**

**Table S3.** BLASTp search for P1 encoded by ORF1 on dsRNA1 of CfCV1.

| Virus name | Family | Genus | Total score | E value | Query cover (%) | Identity (%) | GenBank accession no. |
| --- | --- | --- | --- | --- | --- | --- | --- |
| Botryosphaeria dothidea chrysovirus 1 | *Chrysoviridae* | *Chrysovirus* | 1014 | 0 | 99 | 48 | AGZ84312 |
| Penicillium janczewskii chrysovirus 2 | *Chrysoviridae* | *Chrysovirus* | 1010 | 0 | 99 | 49 | ALO50149 |
| Penicillium janczewskii chrysovirus 1 | *Chrysoviridae* | *Chrysovirus* | 876 | 0 | 99 | 42 | ALO50142 |
| Aspergillus mycovirus 1816 | *Chrysoviridae* | *Chrysovirus* | 831 | 0 | 96 | 43 | ABX79996 |
| Tolypocladium cylindrosporum virus 2 | *Chrysoviridae* | *Chrysovirus* | 743 | 0 | 96 | 39 | CBY84993 |
| Magnaporthe oryzae chrysovirus 1 | *Chrysoviridae* | *Chrysovirus* | 704 | 0 | 99 | 39 | BAJ15133 |
| Magnaporthe oryzae chrysovirus 3 | *Chrysoviridae* | *Chrysovirus* | 691 | 0 | 99 | 39 | BAO20927 |
| Fusarium graminearum dsRNA mycovirus-2 | *Chrysoviridae* | *Chrysovirus* | 607 | 0 | 99 | 35 | ADW08802 |
| Fusarium graminearum mycovirus-China 9 | *Chrysoviridae* | *Chrysovirus* | 597 | 0 | 99 | 35 | ADU54123 |
| Fusarium oxysporum f. sp. dianthi mycovirus 1 | *Chrysoviridae* | *Chrysovirus* | 572 | 0 | 99 | 34 | AKP45145 |
| Agaricus bisporus virus 1 | *Chrysoviridae* | *Chrysovirus* | 429 | 1.15E-128 | 93 | 29 | CAA64144 |
| Verticillium dahliae chrysovirus 1 | *Chrysoviridae* | *Chrysovirus* | 157 | 2.66E-35 | 63 | 25 | ADG21213 |
| Cryphonectria nitschkei chrysovirus 1 | *Chrysoviridae* | *Chrysovirus* | 154 | 2.49E-34 | 78 | 23 | ACT79255 |
| Helminthosporium victoriae 145S virus | *Chrysoviridae* | *Chrysovirus* | 153 | 4.04E-34 | 47 | 26 | AAM68953 |
| Amasya cherry disease associated chrysovirus | *Chrysoviridae* | *Chrysovirus* | 152 | 8.2E-34 | 58 | 25 | CAG77602 |
| Bipolaris maydis chrysovirus 1 | *Chrysoviridae* | *Chrysovirus* | 152 | 1.44E-33 | 47 | 26 | ARM36035 |
| Aspergillus fumigatus chrysovirus | *Chrysoviridae* | *Chrysovirus* | 150 | 3.37E-33 | 81 | 24 | CAX48749 |
| Brassica campestris chrysovirus 1 | *Chrysoviridae* | *Chrysovirus* | 149 | 9.9E-33 | 78 | 24 | AKU48197 |
| Penicillium chrysogenum virus | *Chrysoviridae* | *Chrysovirus* | 145 | 1.09E-31 | 48 | 27 | AF296439 |
| Anthurium mosaic-associated virus | *Chrysoviridae* | *Chrysovirus* | 145 | 1.34E-31 | 46 | 26 | ACU11563 |
| Fusarium oxysporum chrysovirus 1 | *Chrysoviridae* | *Chrysovirus* | 142 | 5.54E-31 | 47 | 26 | ABQ53134 |
| Macrophomina phaseolina chrysovirus 1 | *Chrysoviridae* | *Chrysovirus* | 138 | 2.66E-29 | 45 | 27 | ALD89090 |
| Colletotrichum gloeosporioides chrysovirus 1 | *Chrysoviridae* | *Chrysovirus* | 135 | 2.18E-28 | 47 | 28 | ALW95408 |
| Raphanus sativas chrysovirus 1 | *Chrysoviridae* | *Chrysovirus* | 128 | 3.72E-26 | 46 | 26 | AFE83590 |
| Isaria javanica chrysovirus 1 | *Chrysoviridae* | *Chrysovirus* | 126 | 1.12E-25 | 48 | 24 | APR73428 |
|  |  |  |  |  |  |  |  |
