# Supplementary material for: Identification and Characterization of a Novel Hepta-Segmented dsRNA Virus From the Phytopathogenic Fungus Colletotrichum fructicola
Source: Front Microbiol. 2018 Apr 19;9:754. doi: 10.3389/fmicb.2018.00754 (PMC5917037; doi:10.3389/fmicb.2018.00754)
Supplement: Supplementary file 4 [file Table_4.DOCX]

**Supplementary**

**Table S4.** BLASTp search for P2-P4 encoded by dsRNA2–4 of CfCV1.

| Protein | Virus name | Family | Total score | E value | Query cover (%) | Identity (%) | GenBank accession no. |
| --- | --- | --- | --- | --- | --- | --- | --- |
| P2 | Colletotrichum fructicola chrysovirus 1 |  |  |  |  |  |  |
| putative coat protein (BdCV1-P2) | Botryosphaeria dothidea chrysovirus 1 | *Chrysoviridae* | 444 | 6e-141 | 97 | 35 | AGZ84313 |
| 84 kDa protein (PjCV1-P2) | Penicillium janczewskii chrysovirus 1 | *Chrysoviridae* | 357 | 2e-107 | 98 | 32 | ALO50143 |
| ORF2 (PjCV2-P2) | Penicillium janczewskii chrysovirus 2 | *Chrysoviridae* | 423 | 8e-133 | 86 | 36 | ALO50150 |
| hypothetical protein (MOCV1-A-P4) | Magnaporthe oryzae chrysovirus 1-A | *Chrysoviridae* | 171 | 2e-40 | 49 | 29 | BAJ15136 |
| hypothetical protein (MOCV1-A-P4) | Magnaporthe oryzae chrysovirus 1-B | *Chrysoviridae* | 52 | 3e-34 | 68 | 27 | BAO20930 |
| hypothetical protein (FgV2-P3) | Fusarium graminearum dsRNA mycovirus-2 | *Chrysoviridae* | 173 | 4e-41 | 78 | 27 | ADW08804 |
| putative coat protein (FgV-ch9-P3) | Fusarium graminearum mycovirus-China 9 | *Chrysoviridae* | 168 | 2e-39 | 78 | 27 | ADU54125 |
| putative coat protein (FodCV1-P3) | Fusarium oxysporum f. sp. dianthi mycovirus 1 | *Chrysoviridae* | 164 | 4e-38 | 71 | 28 | AKP45147 |
| ORF (AbV1-L5) | La France disease virus | *Chrysoviridae* | 82.8 | 2e-12 | 62 | 23 | BAA01612 |
| P3 | Colletotrichum fructicola chrysovirus 1 |  |  |  |  |  |  |
| hypothetical protein (BdCV1-P3) | Botryosphaeria dothidea chrysovirus 1 | *Chrysoviridae* | 214 | 4e-56 | 78 | 30 | AGZ84314 |
| 80 kDa protein (PjCV1-P3) | Penicillium janczewskii chrysovirus 1 | *Chrysoviridae* | 123 | 2e-25 | 47 | 31 | ALO50144 |
| ORF3 (PjCV2-P3) | Penicillium janczewskii chrysovirus 2 | *Chrysoviridae* | 168 | 5e-40 | 74 | 30 | ALO50151 |
| hypothetical protein (MOCV1-A-P2) | Magnaporthe oryzae chrysovirus 1-A | *Chrysoviridae* | 60.1 | 1e-05 | 32 | 28 | BAJ15134 |
| hypothetical protein (MOCV1-B-P2) | Magnaporthe oryzae chrysovirus 1-B | *Chrysoviridae* | 58.2 | 6e-05 | 32 | 26 | BAO20928 |
| P4 | Colletotrichum fructicola chrysovirus 1 |  |  |  |  |  |  |
| hypothetical protein (BdCV1-P4) | Botryosphaeria dothidea chrysovirus 1 | *Chrysoviridae* | 400 | 1e-126 | 85 | 40 | AGZ84315 |
| 70 kDa protein (PjCV1-P4) | Penicillium janczewskii chrysovirus 1 | *Chrysoviridae* | 326 | 1e-98 | 91 | 34 | ALO50145 |
| ORF4 (PjCV2-P4) | Penicillium janczewskii chrysovirus 2 | *Chrysoviridae* | 333 | 2e-101 | 91 | 34 | ALO50152 |
| hypothetical protein (MOCV1-A-P3) | Magnaporthe oryzae chrysovirus 1-A | *Chrysoviridae* | 245 | 2e-67 | 80 | 32 | BAJ15135 |
| hypothetical protein (MOCV1-B-P3) | Magnaporthe oryzae chrysovirus 1-B | *Chrysoviridae* | 245 | 2e-67 | 80 | 33 | BAO20929 |
| hypothetical protein (FodCV1-P2) | Fusarium oxysporum f. sp. dianthi mycovirus 1 | *Chrysoviridae* | 208 | 9e-54 | 89 | 29 | AKP45146 |
| hypothetical protein (FgV-ch9-P2) | Fusarium graminearum mycovirus-China 9 | *Chrysoviridae* | 194 | 6e-49 | 83 | 29 | ADU54124 |
| hypothetical protein (FgV2-P2) | Fusarium graminearum dsRNA mycovirus-2 | *Chrysoviridae* | 193 | 2e-48 | 83 | 29 | ADW08803 |
