# Supplementary material for: Identification and Characterization of a Novel Hepta-Segmented dsRNA Virus From the Phytopathogenic Fungus Colletotrichum fructicola
Source: Front Microbiol. 2018 Apr 19;9:754. doi: 10.3389/fmicb.2018.00754 (PMC5917037; doi:10.3389/fmicb.2018.00754)
Supplement: Supplementary file 5 [file Table_5.DOCX]

**Supplementary**

**Table S5.** PMF-MS analysis of p72 encoded by ORF2 of Colletotrichum fructicola dsRNA virus 1.

| Amino acid  position | Calculated  Mass | Observed  Mass | ± delta | Amino acid sequence | Ions score |
| --- | --- | --- | --- | --- | --- |
| 1–23 | 2616.096 | 2616.13 | -0.0336 | MADELFASMVDAQPSLGMHDTFR | 61 |
| 24–85 | 6527.038 | 6527.063 | -0.0248 | ADDVGNAYSASPDFDLSGDGAASQAVAAELLGPLGWELYQSLGPDNTTHVTVQYYLEQEGVR | 92 |
| 86–92 | 849.4233 | 849.4344 | -0.0111 | YTSPNLR | 42 |
| 93–104 | 1309.612 | 1309.615 | -0.0033 | SVDVTYSEPTGR | 75 |
| 105–111 | 946.4487 | 946.4548 | -0.0061 | EVYFAYR | 45 |
| 115–130 | 1744.855 | 1744.857 | -0.002 | MAPFTVSQEAATHSLR | 121 |
| 132–152 | 2316.085 | 2316.096 | -0.0104 | YAVESMTGVPLGAGVEQHHYR | 128 |
| 153–181 | 3143.692 | 3143.705 | -0.0134 | VLQTGLHIESLLTTMWLIHLDVVAGADAK | 92 |
| 182–189 | 899.4743 | 899.4825 | -0.0082 | VNIDQGVR | 61 |
| 190–206 | 1914.975 | 1914.98 | -0.0054 | QALAPGLNLEQERDYAK | 94 |
| 203–212 | 1118.604 | 1118.608 | -0.0048 | DYAKTLPGVR | 30 |
| 213–232 | 2178.155 | 2178.158 | -0.0033 | VHINSLDNVAKALIVAACDR | 83 |
| 224–241 | 1856.97 | 1856.989 | -0.0193 | ALIVAACDRNTLVGASAR | 73 |
| 245–249 | 722.3452 | 722.35 | -0.0048 | YQWAR | 35 |
| 250–275 | 2673.32 | 2673.326 | -0.0058 | VPLTMYGGQLPGAMQVALNSPDATAR | 108 |
| 276–287 | 1348.647 | 1348.652 | -0.0055 | AIVNFADHYGSR | 92 |
| 288–295 | 876.4251 | 876.431 | -0.0059 | VACGMALR | 58 |
| 296–309 | 1600.749 | 1600.759 | -0.0095 | TACMLYGITLNNSK | 91 |
| 310–335 | 2839.351 | 2839.371 | -0.0203 | VVLNFAEPSLHEGTHANGDVMYGLNR | 130 |
| 338–364 | 2893.548 | 2893.577 | -0.0292 | ELAGRELVALAVYLGHTLQQTSGQAIR | 55 |
| 343–364 | 2367.287 | 2367.291 | -0.0043 | ELVALAVYLGHTLQQTSGQAIR | 125 |
| 365–384 | 1993.078 | 1993.096 | -0.0175 | GAVLGLGAKDVPNVENTIAR | 97 |
| 374–384 | 1226.622 | 1226.626 | -0.0031 | DVPNVENTIAR | 74 |
| 385–395 | 1274.632 | 1274.64 | -0.0083 | SQSLLLQNCGR | 61 |
| 397–416 | 2452.116 | 2452.127 | -0.0107 | LHEWWQGACGTYLDVAEYVR | 109 |
| 417–426 | 1180.662 | 1180.668 | -0.0058 | LNLKHTTAQR | 44 |
| 427–453 | 2824.507 | 2824.516 | -0.0089 | GVIHALSVGFVAEGTMLETVSQPITLR | 150 |
| 454–470 | 1678.729 | 1678.744 | -0.0143 | SAADVAVGDDPYGSAER | 100 |
| 454–472 | 1907.88 | 1907.886 | -0.0066 | SAADVAVGDDPYGSAERTK | 8 |
| 473–500 | 2887.438 | 2887.446 | -0.0082 | ASAQWYVVSGLLTDGGESLQHSVGALSR | 127 |
| 501–513 | 1503.873 | 1503.879 | -0.006 | TLGPHVKLHPHIR | 52 |
| 508–513 | 771.445 | 771.4504 | -0.0054 | LHPHIR | 26 |
| 514–539 | 2803.432 | 2803.442 | -0.0099 | HFAGSTVRFVIAGFTAPMHLSMATVR | 40 |
| 522–539 | 1963.989 | 1964.001 | -0.0123 | FVIAGFTAPMHLSMATVR | 110 |
| 540–547 | 948.5066 | 948.5141 | -0.0076 | DVVHQPVR | 46 |
| 540–565 | 2868.457 | 2868.469 | -0.0123 | DVVHQPVRTDDVMDVPIPEGAPEIIK | 68 |
| 548–587 | 4580.125 | 4580.146 | -0.0212 | TDDVMDVPIPEGAPEIIKEVVDQLEPETDSNWLEDMLDQR | 43 |
| 636–652 | 1782.8 | 1782.811 | -0.0111 | GWGPEHTSGEGLLCGAR | 27 |
| 656–668 | 1385.682 | 1385.69 | -0.0082 | QSLDNVAQLDGAR | 115 |
| 681–697 | 1959.86 | 1959.86 | -0.0006 | HCMTPDQQAMASLAEVR | 46 |
| 726–737 | 1379.713 | 1379.723 | -0.0104 | GPLLEHVIESMR | 54 |
| 755–770 | 1816.92 | 1816.932 | -0.0116 | LDNENFTVDQLALGLR | 107 |
| 771–776 | 750.3608 | 750.366 | -0.0052 | QLGDYR | 38 |
