# Supplementary material for: Identification and Characterization of a Novel Hepta-Segmented dsRNA Virus From the Phytopathogenic Fungus Colletotrichum fructicola
Source: Front Microbiol. 2018 Apr 19;9:754. doi: 10.3389/fmicb.2018.00754 (PMC5917037; doi:10.3389/fmicb.2018.00754)
Supplement: Supplementary file 6 [file Table_6.DOCX]

**Supplementary**

**Table S6.** PMF-MS analysis of p68 encoded by ORF4 of Colletotrichum fructicola dsRNA virus 1.

| Amino acid  position | Calculated  Mass | Observed  Mass | ± delta | Amino acid sequence | Ions score |
| --- | --- | --- | --- | --- | --- |
| 2–12 | 1311.6312 | 1311.636 | -0.0049 | SFTVDPAHPWR | 63 |
| 19–27 | 1012.5627 | 1012.5706 | -0.0065 | IPVSHVSFK | 60 |
| 28–49 | 2377.1007 | 2377.1107 | -0.0108 | FSPAQLAEAELVEEEDSAMGVR | 117 |
| 50–66 | 2069.9488 | 2069.9516 | -0.0068 | AWLEHEEPYMLPDDVAR | 74 |
| 67–94 | 2885.3482 | 2885.3613 | -0.0049 | AAYNGEQLQAQSEMTDLGHPQGLASAAK | 114 |
| 95–106 | 1368.6859 | 1368.6925 | -0.0048 | VGTILPYDYNSK | 65 |
| 123–132 | 1056.5366 | 1056.5564 | -0.0198 | AAVNEAVIDR | 105 |
| 123–144 | 2309.0759 | 2309.0813 | -0.0141 | AAVNEAVIDRMNSAMSVDAMAK | 86 |
| 133–144 | 1270.5301 | 1270.5356 | -0.0115 | MNSAMSVDAMAK | 118 |
| 145–152 | 896.5396 | 896.5443 | -0.0062 | DVGLKLPR | 60 |
| 153–173 | 2343.2132 | 2343.2182 | -0.0045 | LVQNAQSKPGDVTYNQLEALR | 100 |
| 174–181 | 948.494 | 948.5029 | -0.012 | YDILQAAR | 50 |
| 182–188 | 879.4024 | 879.4086 | -0.0063 | QDDYALR | 63 |
| 195–206 | 1432.7355 | 1432.7358 | -0.0003 | VVAHSCVLHQQR | 63 |
| 223–254 | 3785.7527 | 3785.7584 | -0.016 | SLLTAELPNITTQELQEDSWWYVGDDLSEDYR | 75 |
| 255–262 | 849.4466 | 849.4531 | -0.0051 | AFLAMAAR | 61 |
| 263–294 | 3670.7371 | 3670.7574 | -0.015 | GLQHFTSAQTDTVYSNCVTEAEVIEQQITFVR | 122 |
| 263–295 | 3798.8539 | 3798.8523 | -0.0062 | GLQHFTSAQTDTVYSNCVTEAEVIEQQITFVRK | 100 |
| 296–311 | 1525.7584 | 1525.7889 | -0.0305 | NGTVPSPQPGGGAFLK | 33 |
| 312–359 | 5158.5774 | 5158.6014 | -0.0141 | VLSSPDLAAAYYYTYAASLGIGHSATQILAHACIGPHLWASEAILPYR | 60 |
| 365–373 | 1018.5732 | 1018.5811 | -0.0071 | LDAGIYLVR | 77 |
| 374–398 | 2693.3945 | 2693.4137 | -0.0206 | ETAVVDQLQLADVQALVNHSAVFAR | 129 |
| 400–412 | 1290.7237 | 1290.7296 | -0.0041 | ALAGLGAVITSYR | 95 |
| 416–425 | 1233.606 | 1233.6136 | -0.01 | KTDVQQTMQR | 98 |
| 417–425 | 1105.5146 | 1105.5186 | -0.0047 | TDVQQTMQR | 93 |
| 426–437 | 1272.6382 | 1272.6496 | -0.0072 | MVGVLAAPEQSR | 76 |
| 442–466 | 2996.5023 | 2996.5079 | -0.0141 | RVHSCLNPGYLGLEWLDPFRPSVER | 71 |
| 443–466 | 2840.4076 | 2840.4068 | -0.0136 | VHSCLNPGYLGLEWLDPFRPSVER | 61 |
| 471–476 | 796.3508 | 796.3538 | -0.0088 | CVEAYR | 43 |
| 477–491 | 1772.9801 | 1772.9798 | -0.0057 | LAHTLLSQFHRPPTR | 51 |
| 495–511 | 1652.8341 | 1652.8444 | -0.0078 | SLFTTGVAMSGVVPGSK | 115 |
| 512–539 | 3054.5123 | 3054.5219 | -0.0106 | AEVASYAELVVYQVLAGEAISCESELVR | 103 |
| 512–560 | 5611.6819 | 5611.7068 | -0.0068 | AEVASYAELVVYQVLAGEAISCESELVRNDFVDDFEPLAMYSHWHALLR | 57 |
| 540–560 | 2591.1739 | 2591.1903 | -0.0113 | NDFVDDFEPLAMYSHWHALLR | 87 |
|  |  |  |  |  |  |
