# Supplementary material for: Identification and Characterization of a Novel Hepta-Segmented dsRNA Virus From the Phytopathogenic Fungus Colletotrichum fructicola
Source: Front Microbiol. 2018 Apr 19;9:754. doi: 10.3389/fmicb.2018.00754 (PMC5917037; doi:10.3389/fmicb.2018.00754)
Supplement: Supplementary file 7 [file Image_1.PDF]

# Supplementary

**Figure S1.** Multiple sequence alignments for the terminal regions of the coding strands of dsRNA1–7 (A), dsRNA1–4 (B), and dsRNA5–7 (C) of CfCV1. Backgrounds that are black, grey, and light grey in color signify nucleotide identity no less than 100%, 80%, and 60%, respectively. The (CAA)n repeats were showed and underlined by red lines. The conserved sequences in the 5'- and 3'-UTRs were emphasized by blue lines. The initiator codons (AUG) of dsRNA1–4 were showed in a green box.

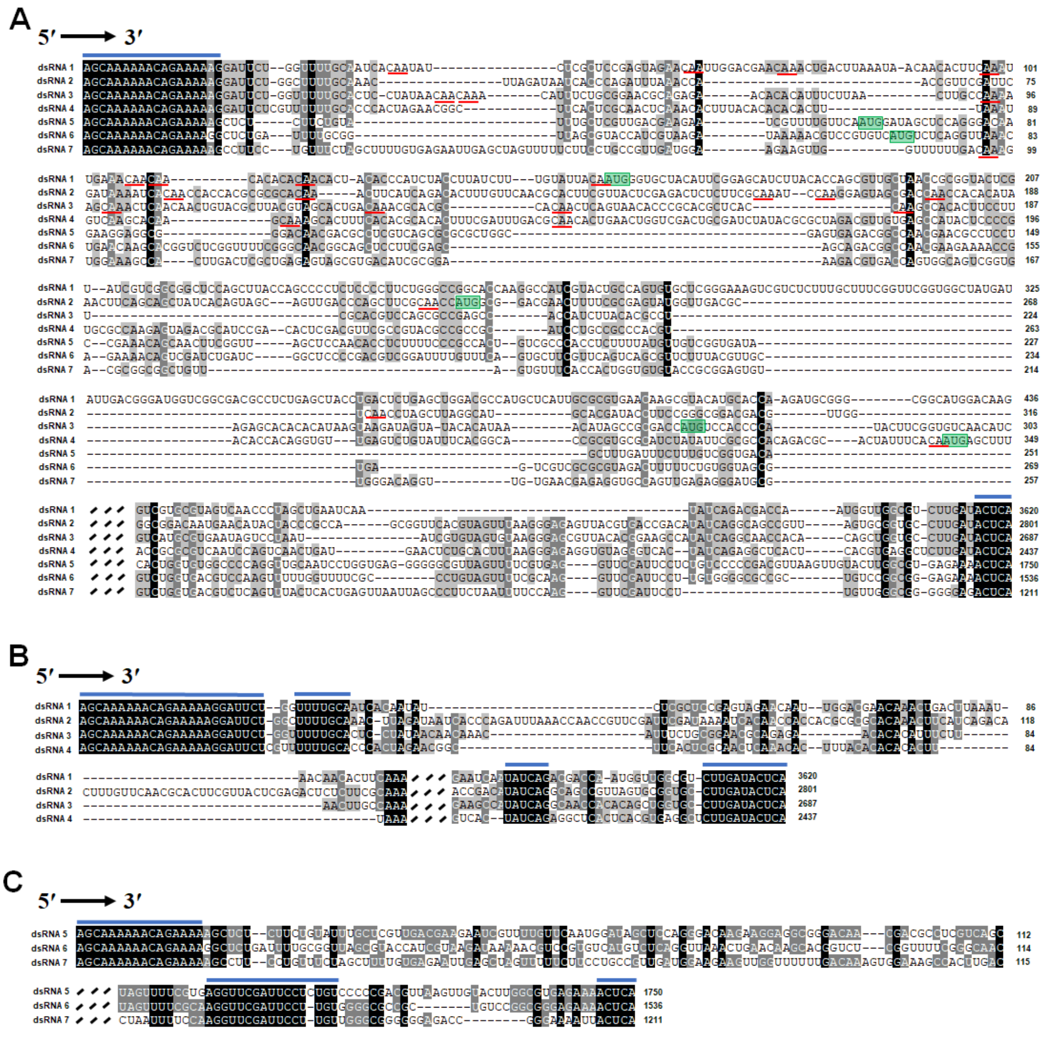


**Supplementary Figure S1**
