# Supplementary material for: Identification and Characterization of a Novel Hepta-Segmented dsRNA Virus From the Phytopathogenic Fungus Colletotrichum fructicola
Source: Front Microbiol. 2018 Apr 19;9:754. doi: 10.3389/fmicb.2018.00754 (PMC5917037; doi:10.3389/fmicb.2018.00754)
Supplement: Supplementary file 8 [file Image_2.PDF]

Supplementary

Figure S2. Agarose gel (1.0%) electrophoresis of the dsRNAs extracted from each fraction of 10 to 40% (wt/vol) sucrose gradient after centrifuged at 70,000×*g* at 4 ℃ for 3 h.

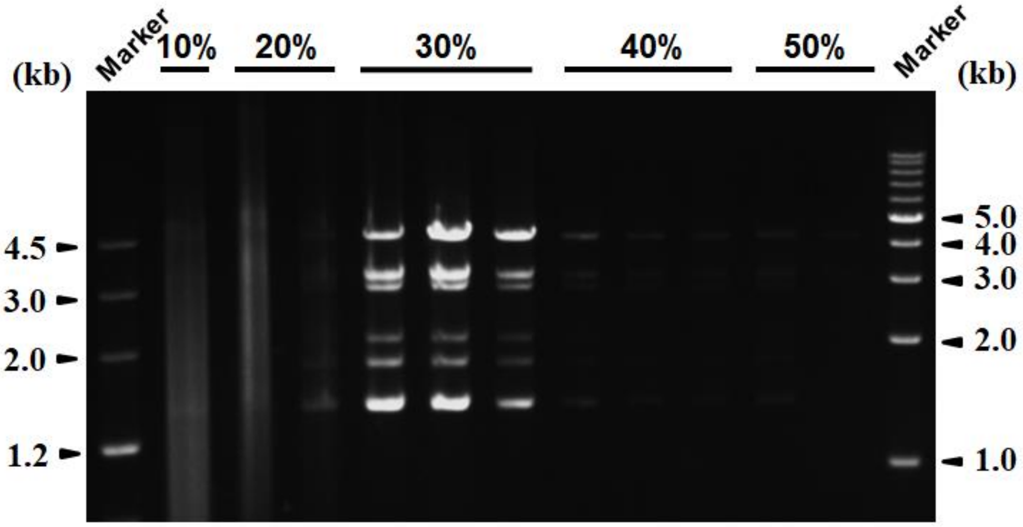

Supplementary Figures S2
