# Supplementary material for: Identification and Characterization of a Novel Hepta-Segmented dsRNA Virus From the Phytopathogenic Fungus Colletotrichum fructicola
Source: Front Microbiol. 2018 Apr 19;9:754. doi: 10.3389/fmicb.2018.00754 (PMC5917037; doi:10.3389/fmicb.2018.00754)
Supplement: Supplementary file 9 [file Image_3.PDF]

Supplementary

Figure S3. RT-PCR confirmation of the CfCV1-infected sub-strains. FJ-4 (labeled with FJ-4) was used as the positive control. FJ-85^{hyp} (labeled with FJ-85^{hyp}) was used as the negative control. The obtained RT-PCR products were cloned and sequenced.

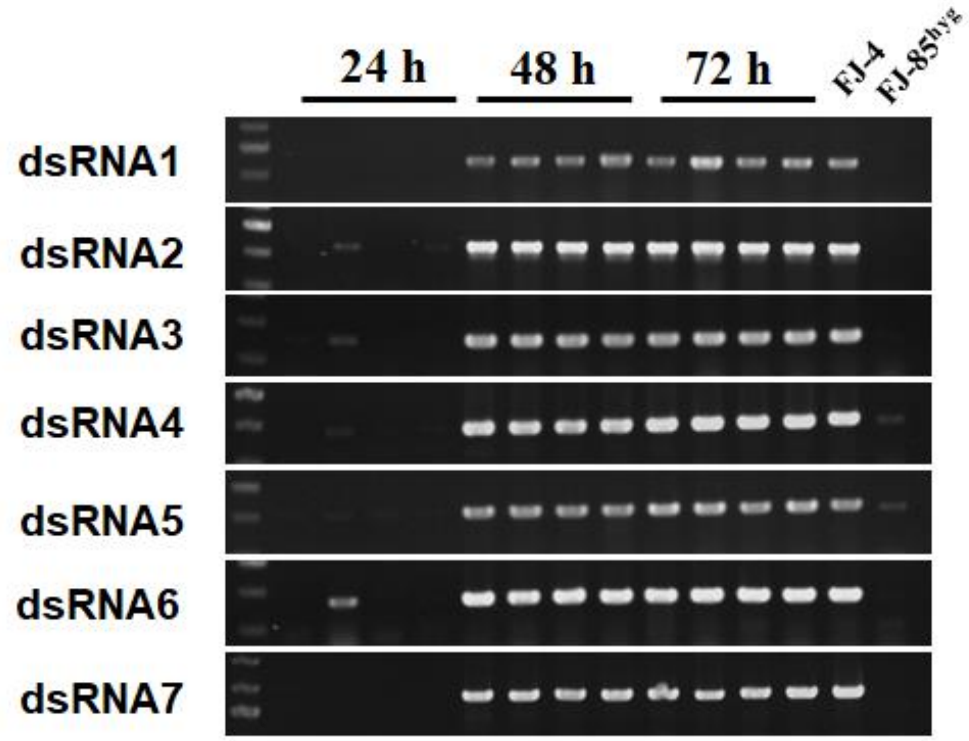

Supplementary Figures S3
