# Supplementary material for: Identification and Characterization of a Novel Hepta-Segmented dsRNA Virus From the Phytopathogenic Fungus Colletotrichum fructicola
Source: Front Microbiol. 2018 Apr 19;9:754. doi: 10.3389/fmicb.2018.00754 (PMC5917037; doi:10.3389/fmicb.2018.00754)
Supplement: Supplementary file 10 [file Image_4.PDF]

# Supplementary

**Figure S4.** Horizontal transmission of CfCV1 from strain FJ-4 to virus-free strains FJ-85 and FJ-4-18. (A) Strain FJ-4 was dual cultured with strains FJ-85 and FJ-4-18. (B) The microcosmic morphological characteristics of hyphal tips of strains FJ-4, FJ-85 and FJ-4-18. The scale bar represents 100 μm.

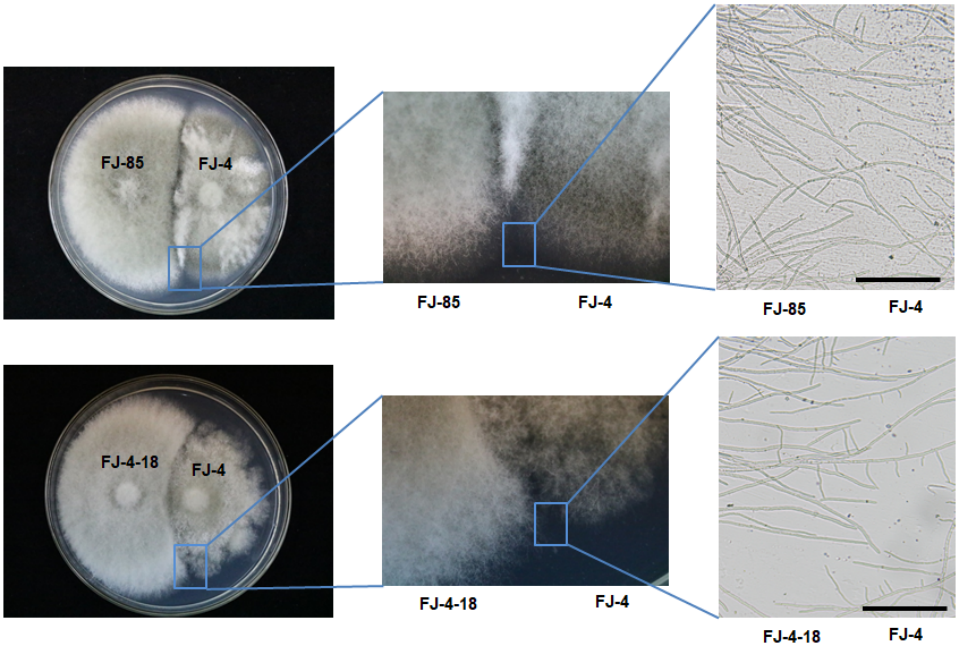


**Supplementary Figures S4**
